# Draft Genome Sequence of Comamonas aquatilis Strain LK (= CSUR P6418 = CECT 9772), Isolated from the Planarian Schmidtea mediterranea

**DOI:** 10.1128/MRA.00297-20

**Published:** 2021-02-04

**Authors:** Luis Johnson Kangale, Anthony Levasseur, Didier Raoult, Eric Ghigo, Pierre-Edouard Fournier

**Affiliations:** a Aix-Marseille University, IRD, AP-HM, SSA, VITROME, Marseille, France; b IHU-Méditerranée-Infection, Marseille, France; c Aix-Marseille University, IRD, AP-HM, MEPHI, Marseille, France; d Special Infectious Agents Unit, King Fahd Medical Research Center, King Abdulaziz University, Jeddah, Saudi Arabia; e Techno-Jouvence, Marseille, France; University of Maryland School of Medicine

## Abstract

Comamonas aquatilis was defined as a new *Comamonas* species based on its 16S rRNA sequence, but the genome from the type strain SB30-Cr27-3^T^ (= CIP 111491^T^ = CCM 8815^T^) is not available. We have cultivated from the planarian Schmidtea mediterranea a Comamonas aquatilis strain, LK (= CSUR P6418 = CECT 9772), that exhibits 100% 16S rRNA sequence similarity to strain SB30-Cr27-3^T^. We have sequenced the genome of strain LK and obtained a chromosome of 4,899,818 bp, with a G+C content of 61.75%, assembled into two contigs.

## Abstract (precis)

Comamonas aquatilis was defined as a new *Comamonas* species based on its 16S rRNA sequence, but the genome from the type strain SB30-Cr27-3^T^ (= CIP 111491^T^ = CCM 8815^T^) is not available. We have cultivated from the planarian Schmidtea mediterranea a Comamonas aquatilis strain, LK (= CSUR P6418 = CECT 9772), that exhibits 100% 16S rRNA sequence similarity to strain SB30-Cr27-3^T^.

## ANNOUNCEMENT

The genus *Comamonas*, assigned to the family *Comamonadaceae* (*Betaproteobacteria*), was originally named by Davis and Park (1) in 1962 but was validly published only in 1985, with Comamonas terrigena as the type species (2). We isolated a bacterial strain, LK (= CSUR P6418 = CECT 9772), from the planarian species Schmidtea mediterranea using a previously published method (3). This strain exhibited 16S rRNA sequence similarity of 100% to Comamonas aquatilis strain SB30-Cr27-3^T^ (= CIP 111491^T^ = CCM 8815^T^); the latter was isolated from a garden pond (4). Because the genomic sequence of the type strain of Comamonas aquatilis (SB30-Cr27-3^T^) was not available, we sequenced the genome from strain LK. Comamonas aquatilis strain LK was grown for 24 h at 28°C on Columbia agar supplemented with 5% sheep blood (bioMérieux, Marcy l’Etoile, France). The genomic DNA (gDNA) of Comamonas aquatilis strain LK was extracted in two steps; first, a mechanical treatment was performed with acid-washed glass beads (G4649-500G; Sigma) using a FastPrep-24 5G grinder (MP Biomedicals) at maximum speed. Then, after a 30-min lysozyme incubation at 37°C, the DNA was extracted using the EZ1 system (Qiagen) with the EZ1 DNA tissue kit. The gDNA of Comamonas aquatilis strain LK was quantified by a Qubit assay (Life Technologies, Carlsbad, CA, USA). The gDNA was then sequenced on the MiSeq platform (Illumina, Inc., San Diego, CA, USA) (5) with the paired-end strategy and was barcoded in order to be used with the Nextera XT DNA sample preparation kit (Illumina). To prepare the paired-end library, dilution was performed to obtain 1 ng of each genome as input. The “tagmentation” step fragmented and tagged the DNA. Then, limited-cycle PCR amplification (12 cycles) completed the tag adapters and introduced dual-index barcodes. After purification on AMPure XP beads (Beckman Coulter, Inc., Fullerton, CA, USA), the libraries were normalized on specific beads according to the Nextera XT protocol (Illumina). Normalized libraries were pooled into a single library for sequencing on the MiSeq system. The pooled single-strand library was loaded onto the reagent cartridge and then onto the instrument along with the flow cell. Automated cluster generation and paired-end sequencing (2 × 250 bp) with dual-index reads were performed in a single 39-hour run. Total information of 10.3 Gb was obtained from a cluster with a density of 1,179,000 clusters/mm^2^, with a cluster passing quality control filters of 89.7%. Within this run, the index representation for Comamonas aquatilis strain LK was determined using an index value of 4.07%. The quality of the 19,945,000 bp from the MiSeq run was checked using FastQC v0.11.8 (6), and the reads were then trimmed using Trimmomatic v0.36.6 (7), with default parameters. Trimmed sequence reads were assembled using the A5 pipeline 20160825 (8) with default parameters. Strain LK was assembled into two contigs (*N*_50_, 4,122,242 bp; *L*_50_, 1) for a total size of 4,899,818 bp, with a G+C content of 61.75% and coverage depth of 32×. The genome annotation was obtained using the Prokka (9) prokaryotic genome annotation software (Galaxy v1.14.5) set with default parameters. A total of 5,807 predicted protein-coding genes were identified, along with 3 complete rRNA operons, 88 tRNAs, and 1 transfer-messenger RNA.

### Data availability.

The complete 16S rRNA gene sequence of Comamonas aquatilis strain LK (= CSUR P6418 = CECT 9772) was deposited in GenBank under accession number LS453311.1. The genome sequence of Comamonas aquatilis strain LK was deposited in GenBank under accession numbers UOOB01000001 and UOOB01000002 (whole-genome shotgun project accession number UOOB01). The raw data from Illumina MiSeq paired-end sequencing were deposited in the SRA under experiment and run accession numbers ERX3992915 and ERR3991497, respectively.
